# Crizotinib and Sunitinib Induce Hepatotoxicity and Mitochondrial Apoptosis in L02 Cells via ROS and Nrf2 Signaling Pathway

**DOI:** 10.3389/fphar.2021.620934

**Published:** 2021-02-01

**Authors:** Lin Guo, Hui Gong, Ting-Li Tang, Bi-Kui Zhang, Lei-Yi Zhang, Miao Yan

**Affiliations:** ^1^ Department of Pharmacy, Second Xiangya Hospital, Central South University, Changsha, China; ^2^ Department of General Surgery, Second Xiangya Hospital, Central South University, Changsha, China

**Keywords:** crizotinib, sunitinib, hepatotoxicity, ROS, Nrf2, mitochondrial apoptosis

## Abstract

Considerable attention has been raised on crizotinib- and sunitinib-induced hepatotoxicity, but the underlying mechanisms need further examination. In addition, limited therapeutic strategies exist to reduce the liver damage caused by crizotinib and sunitinib. This study investigated the mechanisms of crizotinib- and sunitinib-induced hepatotoxicity and the potential mitigation through ROS and Nrf2 signaling. Firstly, crizotinib and sunitinib reduced cell viability in human liver cells (L02 cells) and triggered dramatic liver injury in mice. Subsequently, we found that crizotinib and sunitinib activated the oxidative stress response (decreased level of GPx and SOD, and increased MDA content) *in vivo*. Crizotinib and sunitinib also stimulated hepatocyte mitochondrial apoptosis and necrosis in L02 cells in a dose-dependent manner. *In vivo* studies further confirmed that crizotinib and sunitinib decreased mitochondrial membrane potential and activated apoptosis-associated proteins (cleaved-PARP, cleaved caspase3, cytochrome c, Bcl2 and Bax). Furthermore, mechanistic investigations demonstrated that crizotinib and sunitinib accumulated ROS and inhibited Nrf2 signaling, and that ROS scavenger NAC and Nrf2 agonist tBHQ alleviated the extent of cell damage and the mitochondrial apoptosis during crizotinib- and sunitinib-induced hepatotoxicity in L02 cells. Collectively, these findings indicated that NAC and tBHQ play the crucial roles in crizotinib- and sunitinib-induced mitochondrial apoptosis via the regulation of oxidative stress.

## Introduction

Most cancers therapies were previously dominated by chemotherapy. However, with the advent of the first small-molecule targeted drug imatinib, targeted drugs rapidly developed into the mainstream of anti-tumor drug research with the advantages of significant efficacy, higher safety, less damage to normal cells and improvement of survival and life quality for patients. By the end of 2016, there were 31 small-molecule kinase inhibitors (KIs) for marketing (Shah, 2016; Zhang et al., 2017). Compared to classical chemotherapeutic agents, these KIs have fewer adverse effects, but they also cause new toxicity, such as a significantly increased risk of drug-induced liver injury (DILI) due to long-term administration of the recommended dose of KIs. Studies showed that nearly 20% of FDA-approved KIs carried a "black box warnings" label for rare but potentially life-threatening hepatotoxicity, including sunitinib, and 71% had precautionary warnings of hepatotoxicity, including crizotinib (Zhang et al., 2017). DILI is one of the most common and serious adverse drug reactions (ADR), with severe cases leading to acute liver failure and even death (Fontana et al., 2009).

In 2013, Crizotinib approved by cFDA (China Food and Drug Administration) was the only drug applied to treat locally advanced or metastatic ALK^+^ non-small cell lung cancer with an objective response rate of 50–61% (Blackhall and Cappuzzo, 2016; Metro et al., 2017). In recent years, 10–38% of patients taking crizotinib had elevated transaminases in phase I/II/III clinical trials, and studies showed that transaminases elevation was more common in Chinese patients than in non-Chinese patients (Wang, 2016). A recent meta-study also showed that the use of ALK-tyrosine kinase inhibitors (TKIs) significantly increased the risk of developing all-grade and high-grade hepatotoxicity (Liu et al., 2018). Sunitinib is an oral multitargeted TKI and extends the survival of patients with metastatic renal-cell carcinoma (Kim et al., 2009) and gastrointestinal stromal tumors (Demetri et al., 2009). However, there are many clinical reports on hepatotoxicity caused by sunitinib. According to the European public assessment published by the European medicines agency in 2013, the incidences of all-grade increases in alanine transaminase (ALT)/aspartate transaminase (AST) were about 40–60% and high-grade increases were about 2–5% when patients were treated with sunitinib. And the incidences of all-grade increases in ALT/AST were about 57% and high-grade increases were about 6% when patients were treated with crizotinib (Shah et al., 2013). At present, intervention strategies for liver toxicity of TKIs are generally dose adjustment and discontinuation, and combined with conventional treatment strategies. Notably, some clinical studies have shown that revised dosing still resulted in high toxicity (Powles et al., 2012; Haas et al., 2016). These results, taken together, indicate that the clinical application of crizotinib and sunitinib have been limited due to different degrees of hepatotoxicity.

Studies have shown that liver damage caused by TKIs was mainly related to liver cell damage, rather than cholestasis (Karczmarek-Borowska and Salek-Zan, 2015). In recent years, it has been found that TKIs-triggered hepatotoxicity was not only associated with drug exposure, toxic metabolites, liver transporters, drug metabolism enzyme polymorphism and immune response (Takimoto et al., 2013; Kobayashi et al., 2015; Teo et al., 2015; Ma et al., 2017; Amaya et al., 2018), but also closely related to oxidative stress (Xue et al., 2012), mitochondrial damage and apoptosis (Eno et al., 2016; Chen et al., 2018). However, there still are limited studies on the potential mechanism of TKIs-triggered hepatotoxicity. Moreover, because crizotinib and sunitinib are relatively newer on the market, the research are comparatively fewer, and there are many unknowns.

Recent studies were mainly focused on the direct role of mitochondrial dysfunction and apoptosis on the crizotinib- and sunitinib-induced hepatotoxicity. At concentrations equal to 100-fold maximum concentration (C_max_), mitochondria injury was induced by crizotinib, but not by sunitinib in isolated rat liver mitochondria (Zhang et al., 2017). Another study inversely suggested that crizotinib did not significantly affect mitochondrial functions and inhibited glycolysis only weakly, but induced apoptosis in HepG2 cells (Mingard et al., 2018). Moreover, some findings inversely showed that sunitinib-induced hepatotoxicity was associated with mitochondrial dysfunction and apoptosis of HepG2 cells (Paech et al., 2017; Paech et al., 2018). The divergences can be explained by the different experimental models and experimental condition settings on crizotinib- and sunitinib-induced hepatotoxicity. In addition, crizotinib and sunitinib could significantly increase the reactive oxygen species (ROS) and superoxide dismutase 2 (SOD2) in mitochondria, and reduce glutathione (GSH) levels, indicating that crizotinib and sunitinib can induce mitochondrial oxidative stress, this is, damage to mitochondria is determined (Paech et al., 2017; Zhang et al., 2017; Mingard et al., 2018; Paech et al., 2018). Mitochondria are not only the main sites for ROS production, but also vulnerable to a large accumulation of ROS and other substances (such as Nrf2 activation disorder (Wang et al., 2018; You et al., 2019)) due to the imbalance of oxidative stress. Based on this, we hypothesize whether crizotinib and sunitinib damage mitochondria by inducing oxidative stress, then leading to the death of hepatocytes?

From the above, the exact mechanism of crizotinib- and sunitinib-induced hepatotoxicity merits further investigation. A thorough and systematic study of the role of oxidative stress pathways and mitochondrial apoptosis on the crizotinib- and sunitinib-induced hepatotoxicity is needed, as well as the identification of key signaling proteins and biomarkers, which can provide some effective interventions on hepatotoxicity caused by crizotinib and sunitinib.

In the present study, we used the L02 cell as an *in vitro* model and C57 mice as an *in vivo* model to examine whether oxidative stress and mitochondrial apoptosis was relately to crizotinib- and sunitinib-triggered hepatotoxicity. Then we evaluated the potential protective effects of pretreatment with ROS scavenger n-acetyl-l-cysteine (NAC) and Nrf2 agonist tertiary butylhydroquinone (tBHQ) on the cell viability and mitochondrial apoptotic pathways in L02 cell elicited by crizotinib and sunitinib.

## Materials and Methods

### Drugs and Reagents

Crizotinib (purity ≥ 98%) and sunitinib (purity ≥ 98%) were obtained from Huateng pharmaceuticals-company (Hunan, China). tBHQ and NAC were purchased from Sigma Aldrich (St. Louis, MO, United States) and Solarbio (Beijing, China), respectively. The primary antibodies used in the present study were as follows: anti-Nrf2 (sc-722, Santa Cruz), anti-cleaved caspase3 (c-caspase3) (af7022, Affinity), anti-Bcl2 (ab692, Abcom), anti-Bax (ab32503, Abcom), anti-PARP (bf0719, Affinity), anti-cleaved PARP (af7023, Affinity), anti-cytochrome c (cytc) (ab13575, Abcom). The anti-β-actin (ac006, ABclonal) and anti-Histone H3 (af0863, Affinity) antibody were used as the control.

### L02 Cell Culture

The L02 cells (purchased from Shanghai Zhong Qiao Xin Zhou Biotechnology Co., Ltd.) were cultured at 37°C with 5% CO_2_ in minimum essential DMEM medium (Gibco, Grand Island, NY, United States) supplemented with 10% FBS (Biological Industries, Israel) containing 1% penicillin/streptomycin (Gibco, United States). In all experiments, the cells were plated at an appropriate density according to the experimental design.

### Animal Treatment and Drug Administration

Healthy male C57BL/6J mice, weighting 18–20 g, were purchased from Hunan Slack Jingda Experimental Animal Co., Ltd. (Hunan, China). The animal experiment was approved by the Institutional Animal Care and Use Committee of Central South University (Number:2019sydw0239). The mice were acclimatized for one week and were maintained under a standard conditioned environment, water and normal chow were given ad libitum. The mice were randomly divided into three groups: 1) vehicle control (gavage of 0.5% (w/v) sodium carboxymethyl cellulose (CMC-Na), *n* = 8), 2) crizotinib (*n* = 8, 100 mg/kg/day), 3) sunitinib (*n* = 8, 120 mg/kg/day). Animals were sacrificed after 14 consecutive days, blood samples and livers were collected for further determination.

### Cell Viability Assay

The attached L02 cells were treated with crizotinib (0, 2.5, 5, 10, 15, 20, 30 μM) or sunitinib (0, 2.5, 5, 10, 15, 20, 25, 30 μM) alone for 48 h. Then MTT (Sigma-Aldrich, St. Louis, MO, United States) solution was added to the cells, and then incubated for 3–4 h at 37°C. The absorbance at a wavelength of 490 nm was measured using an automated microplate spectrophotometer (Thermo Multiskan Spectrum, Thermo Electron Corporation, United States).

### Measurement of ALT, AST and Lactate Dehydrogenase (LDH) Leakage

After treatment, the supernatant was collected, the levels of ALT, AST and LDH were measured by the full-automatic clinical analyzer in the laboratory of the second xiangya hospital (7600, HITACHI Ltd., Tokyo, Japan).

### Histopathological Analysis

Liver samples of the mice were fixed in 10% phosphate-buffered formalin and embedded in paraffin. Liver sections were stained with hematoxylin and eosin for histopathological analysis.

### Apoptosis Analysis

To distinguish living cells, apoptotic cells and necrotic cells accurately and count the proportion of each group of cells, it was assayed using FITC Annexin V Apoptosis Detection Kit (Bestbio, Shanghai, China). Briefly, the treated cells for 48 h were digested by trypsin without EDTA and washed with PBS for 3 times strictly. After Annexin V-FITC and PI staining solution were added to the suspended cells, the reaction was incubated in the dark place for 15 min, followed by sample loading and detection through flow cytometry.

### Accumulation of ROS

The intracellular ROS was assayed using the ROS-sensitive fluorescent dye DCFH-DA (Beyotime Biotechnology, Shanghai, China). The treated cells were incubated with 10 µM DCFH-DA at 37°C for 30 min. The fluorescence intensities of stained cells were measured using a fluorescence microplate reader at excitation and emission wavelengths of 488 and 525 nm, respectively.

### Antioxidant Enzymes

With pretreatment of the hepatic tissue, SOD, glutathione peroxidase (GPx) and malondialdehyde (MDA) levels were measured by using corresponding commercial kits (Jiancheng Bioengineering Institute, Nanjing, China) according to the manufacturer’s protocol, respectively.

### Mitochondrial Membrane Potential (MMP)

The MMP was measured with an MMP assay kit with JC-1 (Beyotime Biotechnology, Shanghai, China) according to the manufacturer’s instructions. L02 cells were exposed to different concentrations of crizotinib (0, 5, 10, 15 μM) or sunitinib (0, 5, 10, 15 μM) for 48 h. After JC-1 dyeing solution was added to the culture medium, the hepatocytes were incubated at 37°C for an additional of 20 min, then they were washed with dyeing buffer (1×), and measured immediately by fluorescent microplate reader (Thermo Electron Corporation, USA) at 490/530 nm (JC-1 monomer) and 525/590 nm (JC-1 polymer). Mitochondrial depolarization is specifically indicated by a decrease in the red-to-green fluorescence intensity ratio.

After the liver tissues were resuspended, suspension cells were incubated with JC-1 dyeing at 37 C for 20 min, and then were washed with dyeing buffer (1×) and centrifuged, finally flow cytometry (Beckman, United States) was used to analyze the fluorescence emission. The fluorescence was excited at a wavelength of 488 nm and measured at 525/40 nm (green fluorescence) and 585/42 nm (red fluorescence).”

### Quantitative Real-Time PCR (RT-qPCR)

Total RNA in the liver was extracted with AG RNAex Pro Reagent (Agbio, AG21102). cDNA was synthesized using Evo M-MLV RT Kit (Agbio, AG11705) according to the protocol recommended by the manufacturer. Quantitative RT-qPCR of 4 genes using SYBR Green Premix Pro Taq (Agbio, AG11701) by an LightCycler 96 system (Roche). The Nrf2 primers were: forward 5′-AAG​CAC​AGC​CAG​CAC​ATT​CTC​C-3′ and reverse 5′-TGA​CCA​GGA​CTC​ACG​GGA​ACT​TC-3′. The HO1 primers were: forward 5′-GGT​ACA​CAT​CCA​AGC​CGA​GA-3′ and reverse 5′-GGT​ACA​AGG​AAG​CCA​TCA​CCA-3′. The NQO1 primers were: forward 5′-ACG​ACA​ACG​GTC​CTT​TCC​AG-3′ and reverse 5′-TCC​TCC​CAG​ACG​GTT​TCC​A-3′. The GAPDH primers were: forward 5′-TCA​CCA​TCT​TCC​AGG​AGC​GAG​AC-3′ and reverse 5′-TGA​GCC​CTT​CCA​CAA​TGC​CAA​AG-3′. Fold changes of treatment groups compared to control groups were determined using the 2^-∆∆CT^ method.

### Western Blotting

The drug-treated cells and hepatic tissue samples were washed with PBS and lysed in RIPA lysis buffer (Beyotime, Shanghai, China). The extracts were centrifuged at 12,000 rmp for 10 min at 4°C, and the supernatants were used for western blotting. the cytoplasmic and nuclear proteins were prepared with the subcellular structure cell nucleus and cytoplasmic protein extraction kit (Boster, Hubei, China) according to the manufacturer’s instruction. Protein concentration was measured using the BCA protein assay kit (Boster, Hubei, China). Equal amounts of protein were electrophoresed using an 8–15% SDS-PAGE gel and transferred to PVDF membranes. The membranes were incubated with indicated primary antibodies at 4°C overnight. After washed in TBST, membranes were incubated with the corresponding secondary antibodies conjugated with HRP for 1 h at room temperature, and then washed. Immunoreactive bands were detected using an ECL kit (NCM Biotech, Suzhou, China).

### Statistical Analysis

Statistical analysis was performed using SPSS 20.0 software. And the results were reported as the means ± SE. The significance of differences among multiple means was assessed by the one-way analysis of variance (ANOVA), and comparison between two groups was done with an independent sample *t*-test. *p* < 0.05 was defined as a statistically significant difference.

## Result

### Crizotinib and Sunitinib Exposure Induced Liver Injury *in vitro* and *in vivo*

**FIGURE 1:**
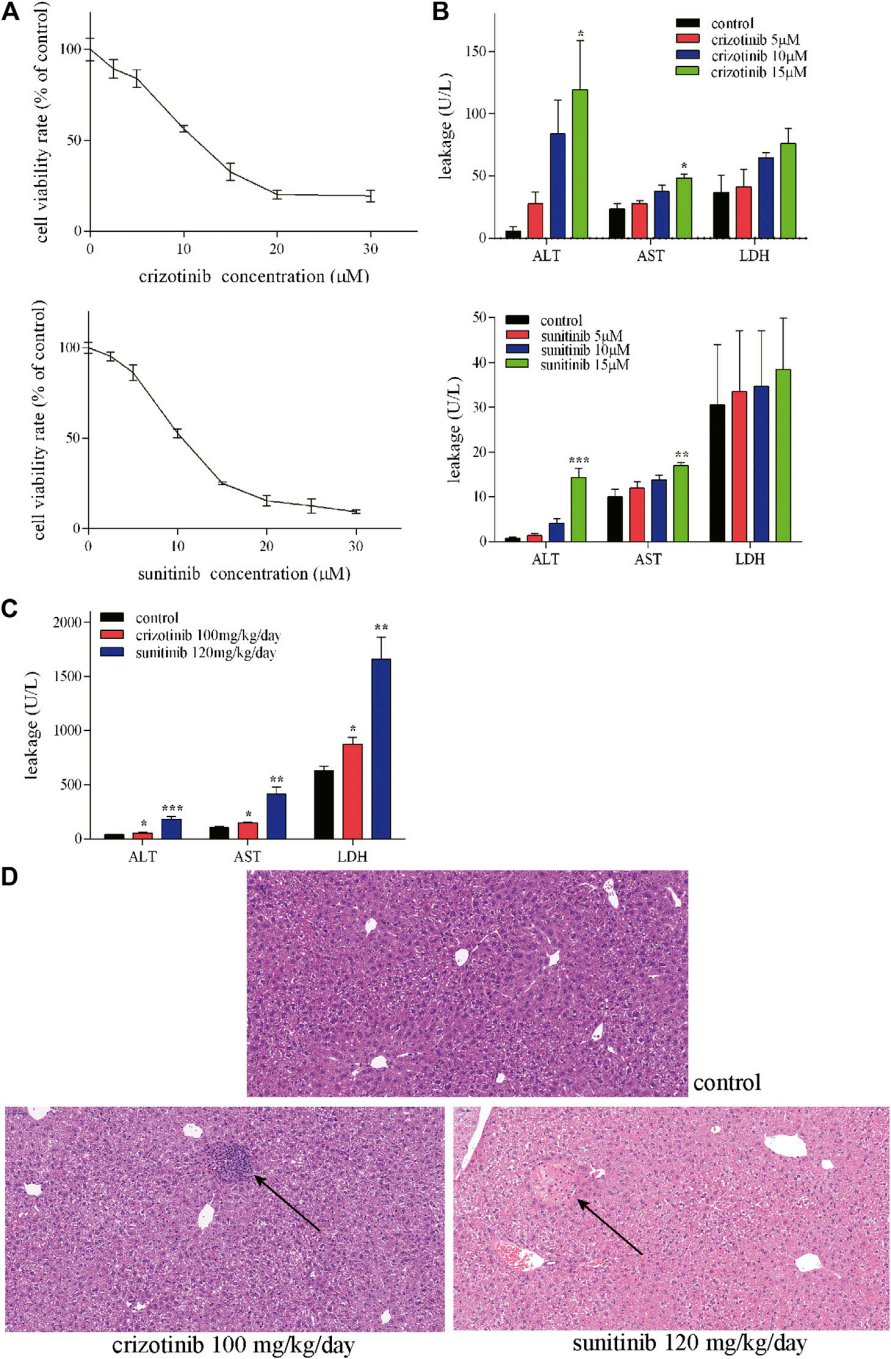
Crizotinib and sunitinib exposure induced hepatotoxicity *in vitro* and *in vivo.* **(A)** Cytotoxicity of crizotinib or sunitinib alone at various concentration for 48 h in L02 cells (*n* = 3). **(B)** ALT, AST and LDH levels in the supernatant following crizotinib or sunitinib exposured at different concentrations for 48 h (*n* = 3). **(C)** Serum ALT, AST and LDH levels of male C57 were determined to evaluate crizotinib- and sunitinib-caused hepatotoxicity (*n* = 5–7). **(D)** Photomicrographs (20×) of hematoxylin & eosin-stained liver sections obtained from the control, crizotinib and sunitinib groups. *p < 0.05, **p < 0.01 and ***p < 0.001 vs. control group.

Crizotinib- and sunitinib-induced hepatotoxicity was examined in human L02 cells because of their similarity to primary hepatocytes (Xu et al., 2020). This cell line is suitable for the pharmacology study due to its retained expression of drug-metabolizing cytochrome P450 enzymes such as CYP3A4 (Huang et al., 2019). To investigate the cytotoxicity induced by crizotinib and sunitinib, the present study assessed the effect of crizotinib or sunitinib treatment with increasing concentrations (0–30 μM) for 48 h. MTT assay showed that both crizotinib and sunitinib treatment reduced cell viability in a dose-dependent manner in L02 cells (Figure 1A). Then the exposure concentrations of 5, 10 and 15 μM for both crizotinib and sunitinib were used for subsequent experiments. A subsequent analysis showed that the ALT and AST levels were elevated in concentration-dependent manner by 48 h exposure of crizotinib and sunitinib, and the biggest rise was observed at the highest concentration (Figure 1B).

Consistent with our L02 cells experiment results, the *in vivo* experiment showed obvious hepatotoxicity. The two weeks of sub lethal dose of crizotinib and sunitinib lead to a remarkable rise of AST, ALT, and LDH levels in the serum in mice compared to the control group (Figure 1C). Moreover, As shown in Figure 1D, there was a nodular lesion near the central vein, with irregular hepatocytes and inflammatory cell infiltration in crizotinib-treated group. And the normal structure of hepatocytes in the lesion disappeared and an eosinophilic lesion around the portal area with partial necrosis and edema of peripheral hepatocytes were found in sunitinib-treated group by the histopathology assessment. These results indicated that both crizotinib and sunitinib induced hepatotoxicity *in vitro* and *in vivo*.

### Crizotinib and Sunitinib Activated Mitochondrial Apoptosis Pathway and Induced Necrosis

**FIGURE 2:**
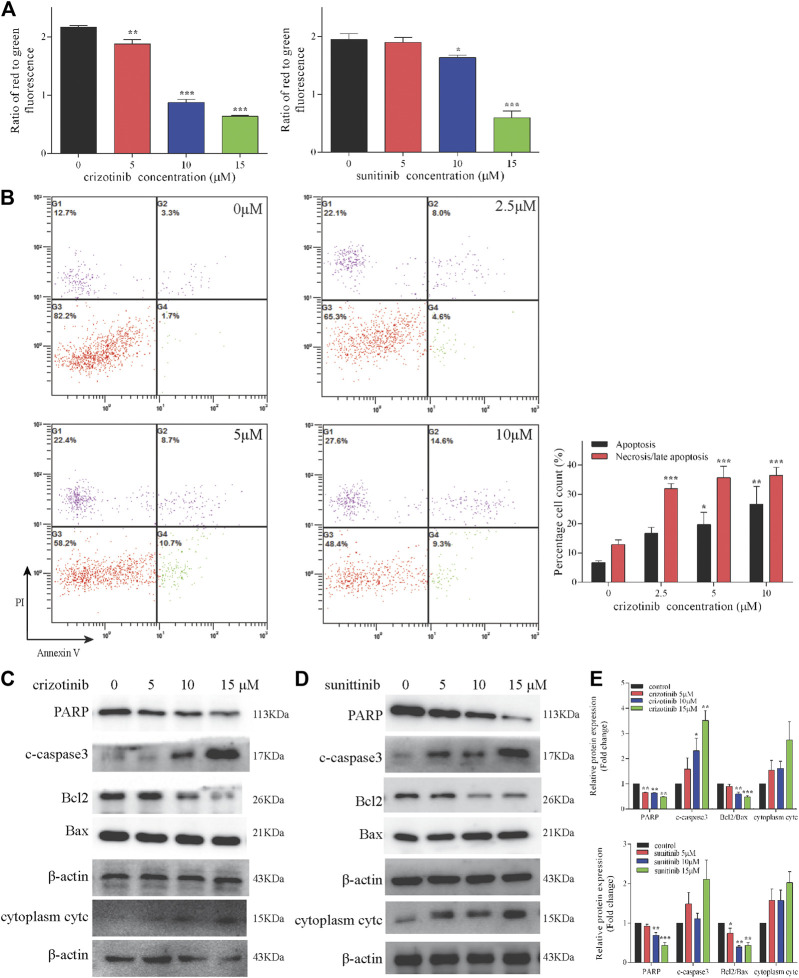
Crizotinib and sunitinib activated mitochondrial apoptosis in L02 cells. **(A)** After treatment with various concentrations of crizotinib or sunitinib for 48 h, the loss of MMP in cells was measured by JC-1 staining (*n* = 3). **(B)** The induction of apoptosis in L02 cells was determined by Annexin V/PI flow cytometry following treatment with increasing concentrations of crizotinib for 48 h, which was also employed to quantify the percentages of apoptotic and necrotic cells (*n* = 4). **(C–E)** The changes of PARP, c-caspase3, Bcl2, Bax and cytc protein expression were observed (*n* = 3). *p < 0.05, **p < 0.01 and ***p < 0.001 vs. control group.

The occurrence and development of many diseases including liver injury are related to apoptosis, in which mitochondrial pathway play an important role. During apoptosis, several key events occur in mitochondria including the depletion of MMP, the release of cytc, altered expression of Bcl2 family protein and activation of caspases cascade. The MMP levels of increasing concentrations of crizotinib and sunitinib depleted markedly as the response increased in L02 cells (Figure 2A). Meanwhile, crizotinib-induced apoptosis was quantified by Annexin V-FITC/PI double staining, demonstrating a concentration-dependent increase in total apoptosis rate and necrosis/late apoptosis rate (Figure 2B). Moreover, we investigated the altered expression of mitochondrial apoptosis-associated proteins. Our subsequent western blot assays demonstrated that crizotinib dose-dependently decreased PARP level and the ratio of Bcl2/Bax, and increased the level of c-caspase3 (Figures 2C,E), consistent with Figure 2B. Cytoplasm cytc level was not statistically increased by crizotinib (Figures 2C,E). Similarly, sunitinib treatment appeared to significantly decrease the PARP and Bcl2/Bax levels, but no significant difference was observed in the c-caspase3 and cytoplasm cytc expression (Figures 2D,E).

**FIGURE 3:**
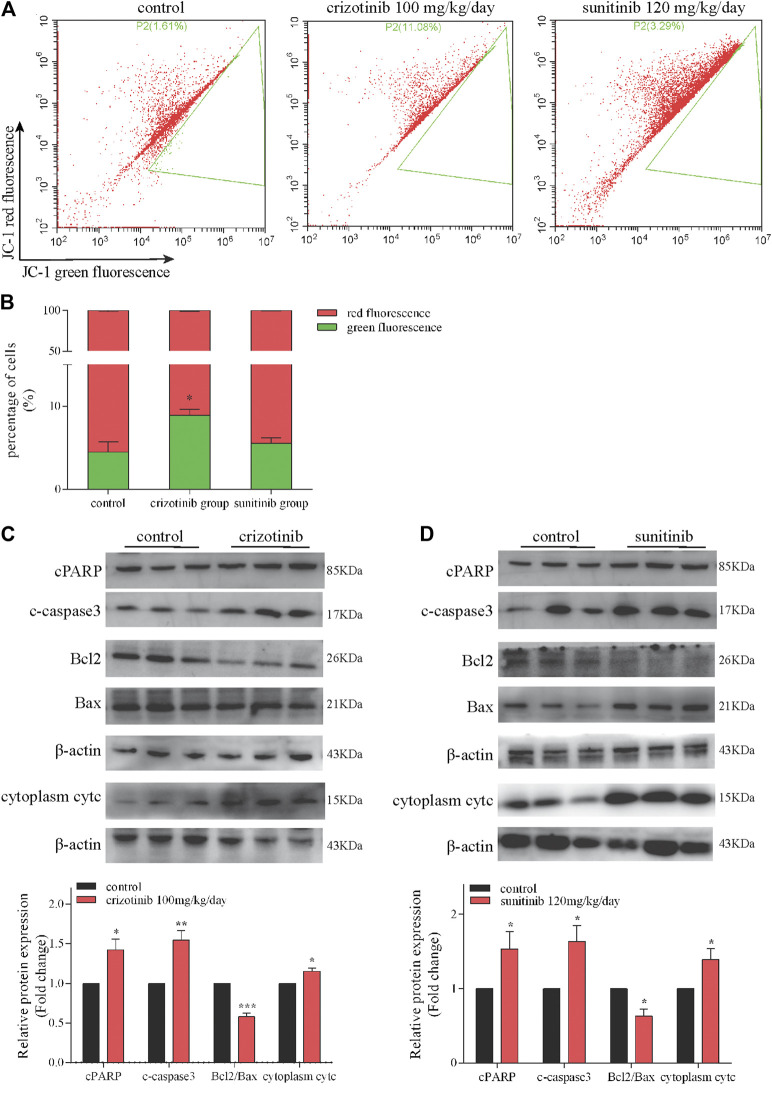
Crizotinib and sunitinib induced mitochondrial apoptosis *in vivo*. **(A, B)** The ratio of red-to green-stained cells after different treatment is shown (*n* = 5–6). In the flow diagram, the abscissa represents the green fluorescence of JC-1, and the ordinate represents the red fluorescence of JC-1, and P2 (xx%) represents xx% of the cells under green fluorescence. **(C, D)** The levels of apoptosis signaling proteins (cPARP, c-caspase3, Bcl2, Bax and cytc) were measured by western blotting (*n* = 6). *p < 0.05, **p < 0.01 and ***p < 0.001 vs. control group.

The results of *in vitro* studies were further validated *in vivo.* Representative images of crizotinib- and sunitinib-induce MMP depletion in livers were showed in Figures 3A,B. The quantification of the relative percentage of red-to-green fluorescent cells significantly decreased in the crizotinib-treated group, but no statistic difference in the sunitinib-treated group compared with control. Also, we detected the protein levels of some key mitochondrial apoptosis-associated protein. The results showed that crizotinib group and sunitinib group both remarkably increased cPARP, c-caspase3 and cytoplasm cytc, and decreased ratio of Bcl2/Bax that might contribute to the loss of MMP (Figures 3C,D).

### Oxidative Stress Played a Crucial Role in Crizotinib- and Sunitinib-Induced Hepatotoxicity *in vitro* and *in vivo*

**FIGURE 4:**
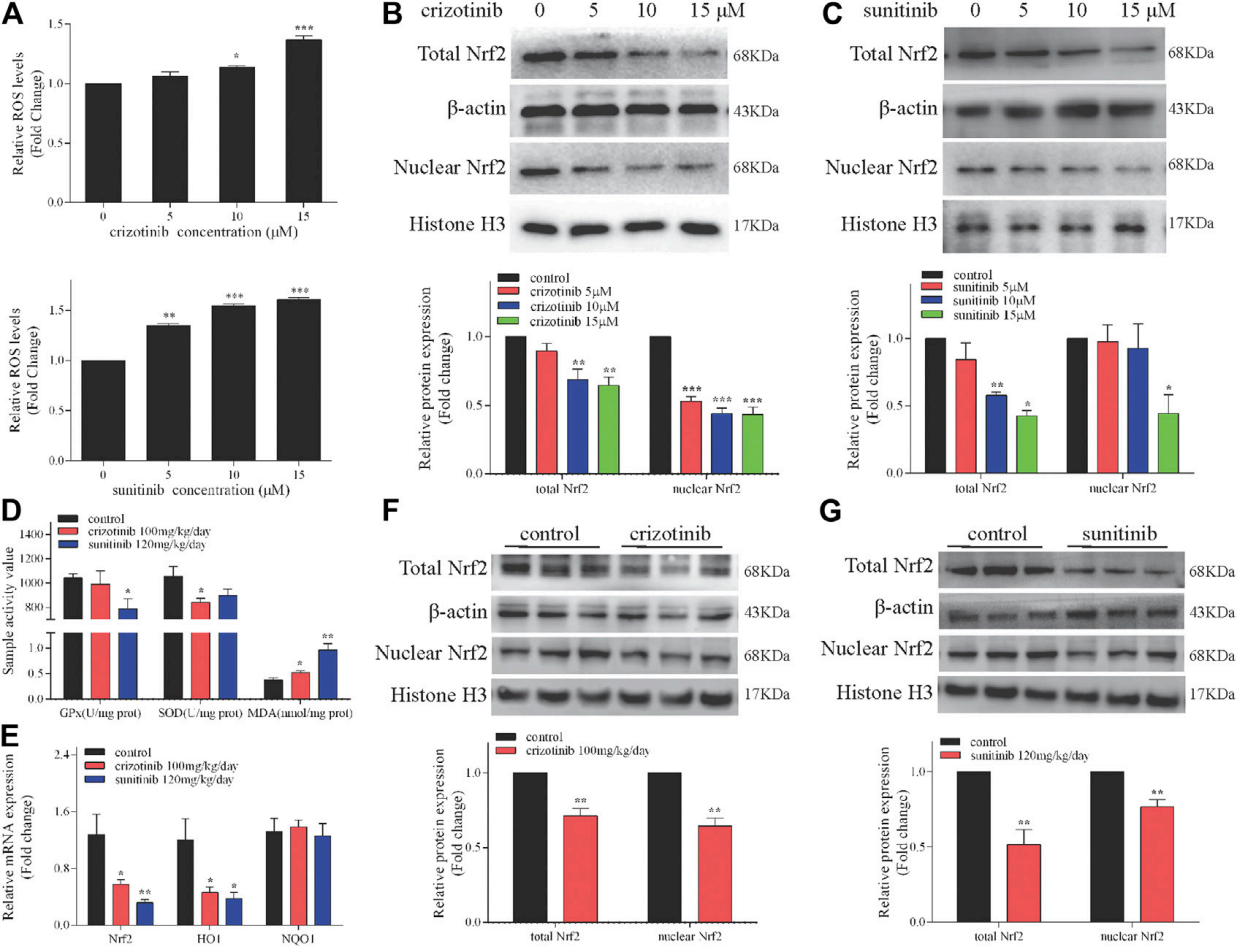
Oxidative stress played a crucial role in crizotinib- and sunitinib-induced hepatotoxicity *in vitro* and *in vivo*. **(A)** L02 cells were incubated with various concentrations of crizotinib or sunitinib for 48 h (*n* = 3). ROS levels were analyzed using a ROS assay kit. **(B, C)** Total and nuclear Nrf2 levels in L02 cells were measured. **(D)** C57 mice were gavaged with 100 mg/kg/day crizotinib or 120 mg/kg/day sunitinib for 14 days (*n* = 6). The levels of GPx, MDA and SOD were measured to assess oxidative stress. **(E)** The mRNA levels of Nrf2, HO1 and NQO1 were determined by RT-qPCR (*n* = 8). **(F,** **G)** The hepatic protein levels of total and nuclear Nrf2 were determined by western blot analysis (*n* = 6). *p < 0.05, **p < 0.01 and ***p < 0.001 vs. control group.

To investigate the effect of crizotinib and sunitinib on the cellular oxidative stress reaction, ROS level was measured in L02 cells using a DCFH-DA probe. As shown in Figure 4A, the relative DCF-positive cells significantly increased after medium to high concentration of crizotinib treatment for 48 h. Similarly, 48 h of sunitinib treatment statistically increased DCF-positive cells in a concentration-dependent manner. After nuclear translocation of Nrf2, then it is combined with the antioxidant response element ARE, which is a key step to activate it and resist oxidative stress. Therefore, western blot was used to detect the changes of total and nuclear Nrf2 protein expression levels after incubation of crizotinib and sunitinib for 48 h. As shown in Figure 4B, compared to the control group, total and nuclear translocation of Nrf2 were dose-dependently inhibited after the crizotinib challenge for 48 h. Meanwhile, total Nrf2 protein was significantly downregulated by medium to high concentration of sunitinib exposure, and nuclear Nrf2 was only dramatically prevented by high sunitinib exposure (Figure 4C).

We further authenticated the effect of crizotinib and sunitinib on the Nrf2 pathway and redox-related indicators *in vivo*. Firstly, we measured levels of GPx, SOD, MDA in the livers. The results showed that crizotinib decreased SOD and induced MDA, but sunitinib significantly reduced the level of GPx and induced MDA (Figure 4D). Then we found that consistent with *in vitro* result, the mRNA level of Nrf2 and HO1 and protein level of total and nuclear Nrf2 downregulated significantly (Figures 4E–G).

### NAC Protected Against Crizotinib- and Sunitinib-Hepatotoxicity *in vitro*

**FIGURE 5:**
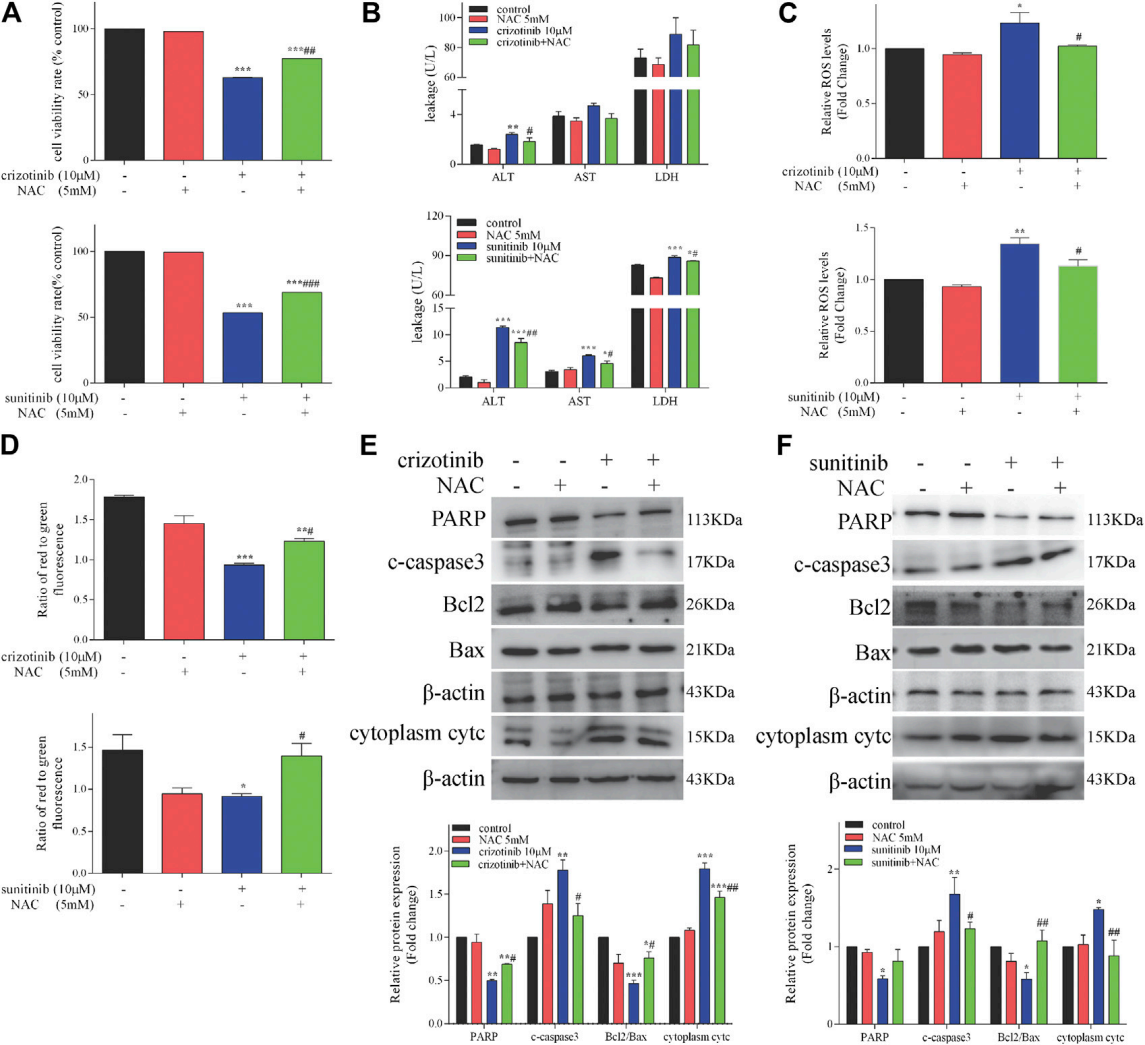
NAC treatment decreased hepatocyte damage and restored mitochondrial apoptosis in L02 cells (n = 3). **(A)** L02 cells were incubated with crizotinib or sunitinib for 48 h in the presence or absence of 5 mM NAC pretreatment for 1 h. Cell viability was assessed using a MTT assay. **(B)** Levels of ALT, AST and LDH released into the supernatant. **(C)** Fold change in ROS level. **(D)** The altered levels of MMP were measured by JC-1 staining. **(E, F)** The levels of apoptosis signaling proteins were analyzed by western blotting. *p < 0.05, **p < 0.01 and ***p < 0.001 vs. control group; ^#^ *p* < 0.05, ^##^ *p* < 0.01 and ^###^ *p* < 0.001 vs. crizotinib or sunitinib group.

As a direct consequence of antioxidant and SH-donating properties, NAC restores cellular redox-status and can in this way modulate the activity of redox-sensitive cell-signaling and transcription pathways (Zafarullah et al., 2003; Sadowska et al., 2007). To assess whether NAC could protect against crizotinib- and sunitinib-induced hepatotoxicity, L02 cells were treated with 10 μM crizotinib (or sunitinib) for 48 h with or without pretreatment with 5 mM NAC. As shown in Figure 5A, the MTT assay showed pretreatment with NAC could significantly restored about 15% of cell viability, compared with the crizotinib (or suinitinib) group. In addition, the release of ALT, AST and LDH were decreased to a less extent by NAC and only ALT showed statistically significance when compared with the crizotinib group. All these markers were remarkably downregulated by NAC when compared with the sunitinib group, indicating alleviation of hepatocyte damage (Figure 5B). As shown in Figure 5C, results verified that NAC significantly reduced the level of ROS produced by crizotinib and sunitinib. In the detection of the MMP, the NAC pretreated samples showed a remarkable increase, implying that NAC might restore MMP via removal of ROS (Figure 5D). Moreover, pretreatment with NAC successfully reversed the mitochondrial apoptosis induction with a significant rise in PARP and ratio of Bcl2/Bax levels, along with a remarkable downregulation in cytoplasm cytc and c-caspase3, compared to those of the crizotinib (or sunitinib) group, as shown in Figures 5E,F.

### tBHQ Alleviated Crizotinib- and Sunitinib-Induced Hepatotoxicity by Inhibiting Mitochondrial Apoptosis

**FIGURE 6:**
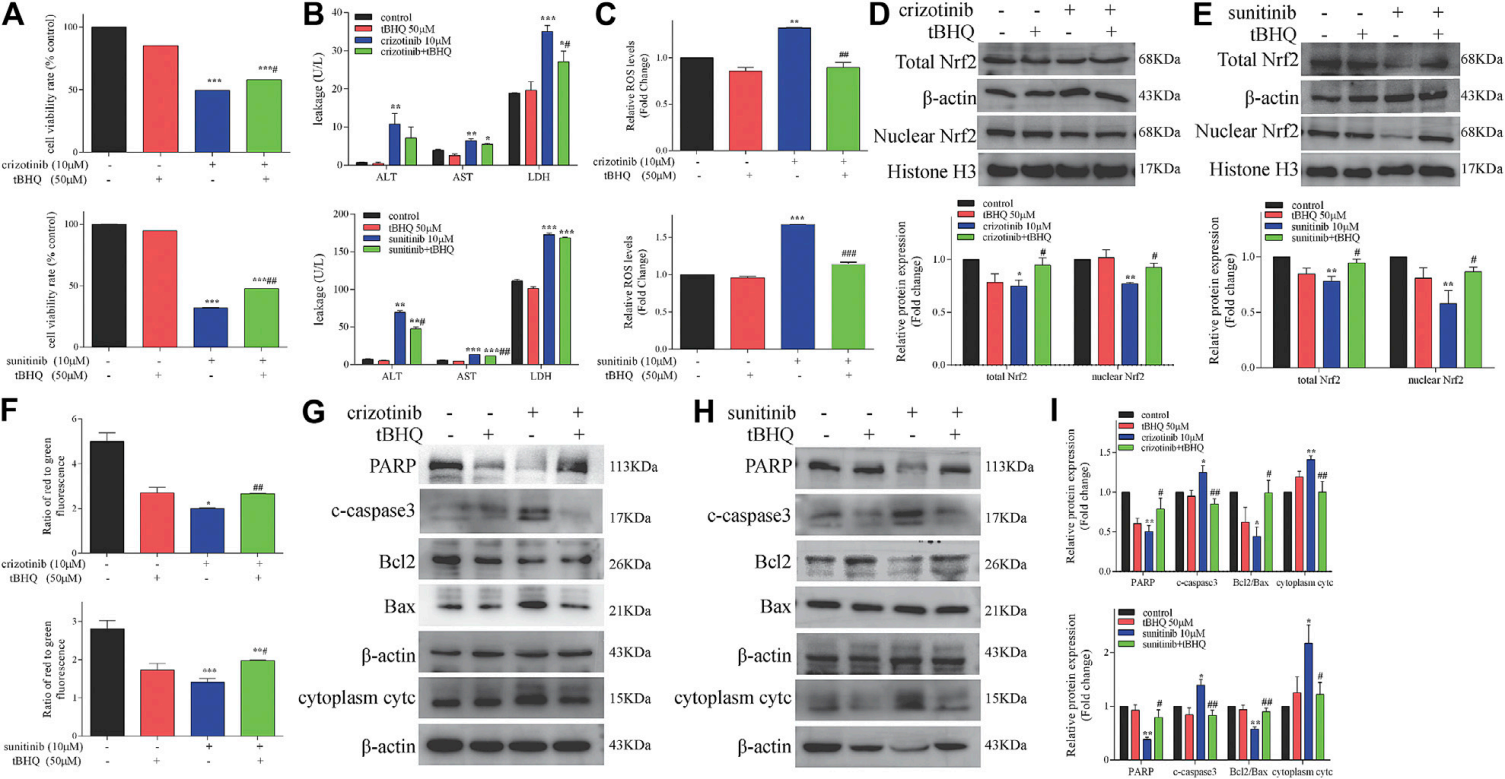
Activation of Nrf2 by tBHQ treatment attenuated the severity of crizotinib- and sunitinib-induced hepatotoxicity and mitochondrial apoptosis. Cells were pretreated with tBHQ (50 μM) for 24 h and co-incubated with crizotinib (10 μM) or sunitinib (10 μM) for 48 h (*n* = 3). **(A)** The viability of hepatocytes was analyzed by performing MTT assays. **(B)** Levels of ALT, AST and LDH released into the supernatant. **(C)** Fold change in ROS level. **(D, E)** Protein expression of total and nuclear Nrf2. **(F)** The altered levels of MMP were measured by JC-1 staining. **(G–I)** Levels of apoptosis-associated proteins were measured by western blotting. *p < 0.05, **p < 0.01 and ***p < 0.001 vs. control group; ^#^ *p* < 0.05 and ^##^ *p* < 0.01 vs. crizotinib or sunitinib group.

We have confirmed that crizotinib and sunitinib exposure induced oxidative stress *in vitro* and *in vivo* (Figure 4), and the Nrf2 signaling pathway is an important pathway for intracellular regulation of oxidative stress response. We next explored whether tBHQ, an antioxidant and the Nrf2 agonist, alleviated crizotinib- and sunitinib-induced hepatotoxicity and mitochondrial apoptosis effectively. L02 cells were pretreatment with or without tBHQ for 24 h and then treated with or without crizotinib (or sunitinib) for 48 h. As shown in Figure 6A, The MTT assay results showed that tBHQ pretreatment enhanced the survival rate of cells by approximately 10% and 15%, respectively, compared with crizotinib or sunitinib treatment alone. Moreover, crizotinib-induced supernatant LDH upregulation were moderately decreased after tBHQ pretreatment, while ALT and AST enhancement was not significantly attenuated (Figure 6B). In addition, only sunitinib-induced supernatant ALT and AST were remarkably decreased after tBHQ pretreatment (Figure 6B). At the same time, we found that tBHQ could effectively reduce ROS induced by the two drugs (Figure 6C). Western blot analysis demonstrated that pretreatment with tBHQ statistically increased the expression of total and nuclear Nrf2 proteins (Figures 6D,E). Meanwhile, activation of Nrf2 expression with tBHQ pretreatment significantly restored MMP (Figure 6F) and alleviated the expression of mitochondrial apoptosis-associated proteins (Figures 6G–I). Taken together, these data confirmed that tBHQ could inhibit the mitochondrial apoptosis, and protect hepatocytes from drug toxicity.

## Discussion

Crizotinib- and sunitinib-induced hepatotoxicity has been well documented by various clinical studies. However, focused studies are limited on the precise mechanism of crizotinib- and sunitinib-induced hepatotoxicity. Also, no treatment is available to mitigate crizotinib- and sunitinib-induced hepatoxicity. The present study demonstrated that crizotinib and sunitinib exposure significantly damaged hepatocytes, induced oxidative stress and mitochondrial apoptosis in L02 cell and mice livers. Moreover, pretreatment with NAC and tBHQ alleviated crizotinib- and sunitinib-induced cellular damage and mitochondrial apoptosis.

Crizotinib and sunitinib not only suppressed the cellullar survival and proliferation of L02 cells but also enhanced the release of ALT and AST, indicating their potential toxicity to the human liver. To date, many studies have supported that crizotinib (Mingard et al., 2018; Yan et al., 2019) and sunitinib (Teo et al., 2015; Bouitbir et al., 2019; Xiao et al., 2019) induced various toxic reactions in a concentration- and time-dependent manner, including hepatotoxicity. In our study, we examined the hepatotoxicity of crizotinib and sunitinib on the C57 mice, which are widely used as an experimental animal model of physiology and pathology. We have previously established an ICR mice model of hepatotoxicity induced by crizotinib and sunitinib (the article is under review). And the emphasis of which was to explore the occurrence of hepatotoxicity and the alters of some proteins associated with hepatotoxicity by mimicking the clinical administration regimen including dose and administration cycle. However, The main purpose of the C57 mice model was to investigate the role of oxidative stress and mitochondrial apoptosis in high doses of crizotinib- and sunitinib-triggered hepatotoxicity. We chose these high doses of exposure because exposing fully the toxicity of crizotinib and sunitinib could reveal the mechanism of toxicity and provide the experimental evidence for alleviating side effects. So far, limited animal models have been established to explore the mechanism of crizotinib- and sunitinib-induced hepatotoxicity (Paech et al., 2018). Thus, we firstly carried out a preliminary dosing experiment. The mice gavaged with crizotinib were assigned to three dosage groups: 100, 250 and 500 mg/kg/day. The mice in the 250 and 500 mg/kg/day group successively died within one week after administration of crizotinib, while the survival rate of the 100 mg/kg/day group was very high within 2 weeks. Therefore, for the subsequent study, we chose this dose, which have been rarely used for research on the mechanism of hepatotoxicity. In contrast, all the mice at the three-dose (40, 80 and 120 mg/kg/day) of sunitinib intragastrical administration survived for 2 weeks. We only observed gradual weight loss in the highest dose group. The dose of sunitinib was previously reported to be 7.5–40 mg/kg/day for 2–4 weeks for most chronic toxicity study (Bouitbir et al., 2019; Kerkela et al., 2009; O'Farrell et al., 2019; Paech et al., 2018).

Oxidative stress is caused by the imbalance between the production of ROS and the clearance of ROS by the antioxidant defense system. When the production of ROS exceeds the antioxidant capacity, it leads to oxidative damage and resulting liver damages. In our study, when treated with crizotinib or sunitinib, L02 cells had increased ROS level and mice liver exhibited an increase of MDA along with the decrease of GPx and SOD, indicating an oxidative stress state. Furthermore, Nrf2 is a key transcription factor that regulates cellular resistance to allogenic substances and oxidative damage (Sykiotis et al., 2011). Activation of Nrf2 pathway can effectively protect the liver from oxidative stress, but the loss or activation disorder of Nrf2 can aggravate the cytotoxicity of oxidative stress, leading to cellular dysfunction, apoptosis, and even cell death. Our previous research showed that when HepG2 cells were exposed to crizotinib and sunitinib for 24 h, nuclear Nrf2 expression enhanced, consistent with the finding in ICR mice administrated with a clinical dose of crizotinib and sunitinib for 4 weeks (the article is under review). On the contrary, in this study, crizotinib and sunitinib decreased total and nuclear Nrf2 expression in L02 cell after 48 h in a concentration-dependent manner as well as in C57 mice at sub lethal dose after 2 weeks, indicating that excessive oxidative stress was induced, which in turn damaged hepatocytes. The difference of Nrf2 expression changes can be explained by different administration dosage and duration of crizotinib or sunitinib treatment, experimental model (such as cell lines, animal species), and property of Nrf2 (Vaziri et al., 2015)***.*** We will identify and further examine dose- or time-dependent dimorphism of Nrf2 expression caused by crizotinib and sunitinib.

Various cellular activities of liver cells are dependent on mitochondria, which not only synthesize ATP to provide energy for cells, but also regulate apoptosis. The main pathway to induce apoptosis, an autonomously ordered and genetically programmed cell death process, is the mitochondria-mediated apoptosis, also known as endogenous pathways. Activation of caspase3 is considered to be one of the key steps in the execution of apoptosis, and PARP is a cleaving substrate of the caspase family and an important indicator for detecting apoptosis. In this study, the analysis showed that the treatment of L02 cells with crizotinib and sunitinib significantly upregulated the c-caspases3, and decreased the PARP levels and the Bcl2/Bax ratio. Meanwhile, crizotinib and sunitinib depleted the MMP, with the release of cytc from the mitochondria into the cytosol. These results demonstrated that mitochondrial apoptosis might play an important role in crizotinib- and sunitinib-induced hepatotoxicity.

Interestingly, the necrosis of L02 cells induced by crizotinib was also distinct and worthy of attention. This result is consistent with that of a recent study in L02 cells (Yan et al., 2019). Is cell necrosis one of the main causes of liver damage caused by crizotinib? At present, studies have found that cell necrosis may be one of the death procedures of hepatotoxicity induced by TKIs. When 10 μM sorafenib was applied to HepG2 cells for 24 h, the total apoptosis rate and necrosis rate increased significantly, suggesting that cell necrosis is also one of the hepatocyte death (Mingard et al., 2018). In addition, regorafinib induced decoupling of mitochondrial respiratory in rat primary hepatocytes, leading to time- and concentration-dependent hepatocyte necrosis (Weng et al., 2015). However, studies (Mingard et al., 2018) showed that when 10 μM and 20 μM crizotinib were incubated in HepG2 cells for 24 h, the total apoptosis rate increased significantly, but the late apoptosis/necrosis rate decreased slightly, showing no statistical significance. In summary, crizotinib can not only induce apoptosis, but also lead to overt cell necrosis. The role of cell necrosis in crizotinib-induced hepatotoxicity will be further explored and related upstream factors will be sought.

The accumulation of ROS can directly attack the mitochondria, reduce the MMP level, damage the mitochondrial function, and then lead to apoptosis. Moreover, mounting evidence indicated that Nrf2 regulates Bcl2 expression and apoptotic cell death (Niture and Jaiswal et al., 2012; Pihan et al., 2017), and the oxidative stress may be a key event to identify mitochondrial apoptosis induced by drugs through ROS or Nrf2 pathway (Gong et al., 2019; Zhang et al., 2019; Kim et al., 2020). ROS accumulation or Nrf2 activation disorders can cause different degrees of damage, which is closely related to cell line, dose and exposure time. In general, severe oxidative stress can eventually lead to cell death through apoptosis or necrosis (Martindale and Holbrook, 2002).

**FIGURE 7:**
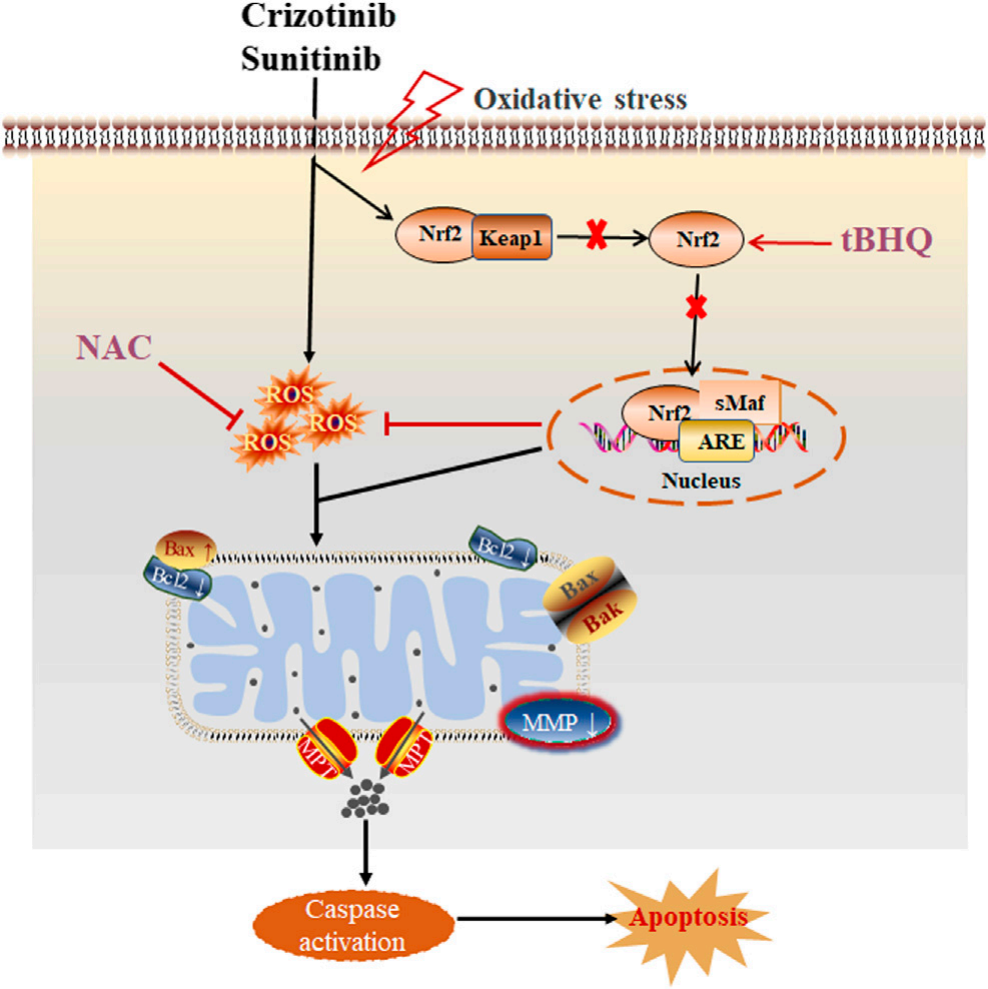
The proposed schematic illustration of crizotinib- and sunitinib-induced liver injury, and tBHQ and NAC combined treatment protecting against crizotinib- and sunitinib-hepatotoxicity.

At present, the researches on the mechanism of TKIs-related hepatotoxicity focus more attention on the changes of mitochondrial oxidative stress to determine the relationship between mitochondrial damage and hepatotoxicity, but less on the important role of oxidative stress in the hepatotoxicity induced by TKIs. However, oxidative stress has been proved to be a significant cause of liver injury. A study (Xue et al., 2012) found that accumulation of ROS, decreased activity of antioxidant enzymes and increase content of MDA when primary hepatocytes were treated with dasatinib. In addition, stress activation of Nrf2 and induction of mitogen activated protein kinases (MAPK) pathway, and pretreatment with NAC could alleviate the degree of liver damage, indicating that oxidative stress is the source of hepatotoxicity caused by dasatinib. Moreover, accumulation of ROS has also been found to be related to apoptosis (Yan et al., 2019). Our data showed that pretreatment with NAC and tBHQ could increase the cell viability and the nuclear translocation of Nrf2, reduce the release of ALT, AST and LDH and ROS level in L02 cells treated with crizotinib and sunitinib, indicating the cellular damage were partially alleviated. In addition, the degree of mitochondria damage and apoptosis was downregulated after pretreatment with NAC and tBHQ. Collectively, these results demonstrated that intracellular oxidative stress (ROS and Nrf2 pathway) might be one of the main causes of crizotinib- and sunitinib-triggered mitochondrial apoptosis. And the proposed mechanism of crizotinib- and sunitinib-induced hepatotoxicity is shown in Figure 7. Accumulation of ROS or the prevention in nuclear translocation of Nrf2 damages oxidative stress and mitochondrial function, finally leads to apoptosis (Pihan et al., 2017). Understanding the molecular toxicological mechanism of crizotinib and sunitinib will be beneficial for the development of new strategies to prevent or alleviate crizotinib- and sunitinib-induced hepatotoxicity.

## Conclusion

In conclusion, our *in vivo* and *in vitro* results demonstrated that crizotinib and sunitinib induced oxidative stress and mitochondrial apoptosis, and finally led to hepatotoxicity. This study provided the evidence that crizotinib and sunitinib induced mitochondrial apoptosis through ROS and Nrf2 pathway in human hepatocytes. These observations offered novel insights into the potential hepatotoxicity of crizotinib and sunitinib as well as similar TKIs.

## Data Availability Statement

The raw data supporting the conclusions of this article will be made available by the authors, without undue reservation.

## Ethics Statement

The animal study was reviewed and approved by Experimental Animal Ethics Committee of Central South University.

## Author Contributions

LG and HG: Contributed to study design and data interpretation, performed lab work and helped to write paper. T-LT and B-KZ: Helped to advised lab work and commented final version of manuscript. L-YZ and MY: Helped in conceptualization, study design, data curation, manuscript writing and resources.

## Funding

This work was financially supported by the National Natural Science Foundation of China (Grant No. 81974532 and 81803830) and Science and Technology Department of Hunan Province, China (Grant No. 2017SK1030).

## Conflict of Interest

The authors declare that the research was conducted in the absence of any commercial or financial relationships that could be construed as a potential conflict of interest.
